# New Fungus-Insect Symbiosis: Culturing, Molecular, and Histological Methods Determine Saprophytic Polyporales Mutualists of *Ambrosiodmus* Ambrosia Beetles

**DOI:** 10.1371/journal.pone.0137689

**Published:** 2015-09-14

**Authors:** Li You, David Rabern Simmons, Craig C. Bateman, Dylan P. G. Short, Matthew T. Kasson, Robert J. Rabaglia, Jiri Hulcr

**Affiliations:** 1 School of Forest Resources and Conservation, Institute of Food and Agricultural Sciences, University of Florida, Gainesville, Florida, United States of America; 2 Department of Entomology and Nematology, Institute of Food and Agricultural Sciences, University of Florida, Gainesville, Florida, United States of America; 3 Division of Plant and Soil Sciences, West Virginia University, Morgantown, West Virginia, United States of America; 4 Forest Health Protection, State & Private Forestry, United States Department of Agriculture Forest Service, Washington, District of Columbia, United States of America; Georg-August-University Goettingen, GERMANY

## Abstract

Ambrosia symbiosis is an obligate, farming-like mutualism between wood-boring beetles and fungi. It evolved at least 11 times and includes many notorious invasive pests. All ambrosia beetles studied to date cultivate ascomycotan fungi: early colonizers of recently killed trees with poor wood digestion. Beetles in the widespread genus *Ambrosiodmus*, however, colonize decayed wood. We characterized the mycosymbionts of three *Ambrosiodmus* species using quantitative culturing, high-throughput metabarcoding, and histology. We determined the fungi to be within the Polyporales, closely related to *Flavodon flavus*. Culture-independent sequencing of *Ambrosiodmus minor* mycangia revealed a single operational taxonomic unit identical to the sequences from the cultured *Flavodon*. Histological sectioning confirmed that *Ambrosiodmus* possessed preoral mycangia containing dimitic hyphae similar to cultured *F*. cf. *flavus*. The *Ambrosiodmus*-*Flavodon* symbiosis is unique in several aspects: it is the first reported association between an ambrosia beetle and a basidiomycotan fungus; the mycosymbiont grows as hyphae in the mycangia, not as budding pseudo-mycelium; and the mycosymbiont is a white-rot saprophyte rather than an early colonizer: a previously undocumented wood borer niche. Few fungi are capable of turning rotten wood into complete animal nutrition. Several thousand beetle-fungus symbioses remain unstudied and promise unknown and unexpected mycological diversity and enzymatic innovations.

## Introduction

Ambrosia beetles (Coleoptera: Curculionidae: Scolytinae and Platypodinae) are a polyphyletic collection of clades within the hyper-diverse bark beetles. Ambrosia beetles have evolved the symbiotic evolutionary mode referred to as fungiculture or fungal farming. The more than 3200 known ambrosia beetle species gain nutrition from fungi grown on abundant yet recalcitrant substrates—dead trees and tree parts. The beetles transport inoculum of specific fungal symbionts from their natal galleries to newly established galleries in a storage organ called a mycangium [1]. Location and morphology of mycangia vary across species and include preoral, mandibular, elytral, mesothoracic, and many others [2], and, in most instances, are phylogenetically highly conserved [1,3]. The only fungal symbionts of ambrosia beetles confirmed thus far have been ascomycotan fungi (Ascomycota: Sordariomycetes) that are transported as budding yeast-like pseudo-mycelium or conidia [4]. In all studied cases, fungi are ecologically “early colonizers”, specialized on extracting nutrients from freshly dead wood but lacking any significant cellulolytic capability [5] and most ambrosial beetle-fungus couples colonize recently killed (dead) trees.

Ambrosia beetles are becoming increasingly economically and ecologically important. Numerous non-native species were inadvertently introduced to new regions via international trade, and in several such cases, formerly harmless beetle species turned into invasive pests [6–8]. In at least four cases, ambrosia beetle-fungus symbionts have triggered acute and ongoing epidemics among susceptible host trees [8–12].

Ambrosia beetles are derived from bark beetles (Coleoptera: Curculionidae: Scolytinae). Bark beetles colonize and consume phloem, a tissue that is more nutrient-rich than wood. Bark beetles, like ambrosia beetles, are also often associated with fungal symbionts, usually ascomycotan and rarely basidiomycotan fungi, and the intensity of association is more variable, ranging from facultative to obligate [13–15]. Beetle-fungus consortia are responsible for spectacular outbreaks, driven either by climate, the pathogenicity of the fungus, or the mass-attacking nature of the beetles [9]. Other insects also engage in symbioses functionally analogous to the ambrosia beetle-fungus symbiosis: attine ants [16], termites [17], ship timber beetles [5], and wood wasps [18]. While most of these use basidiomycotan fungi to gain access to nutrients from plant matter, the fungi are usually either not particularly competitive wood-decomposers or provide only partial nutrition [18]. Though research focused on wood borer-fungus associations has recently increased, it has been almost entirely restricted to a minority of species that happen to be economically important. At the same time, thousands of beetle species remain unstudied, and each new exploration of the understudied taxa revealed interactions with unsuspected and undescribed fungal communities [19,20].

**Fig 1:**
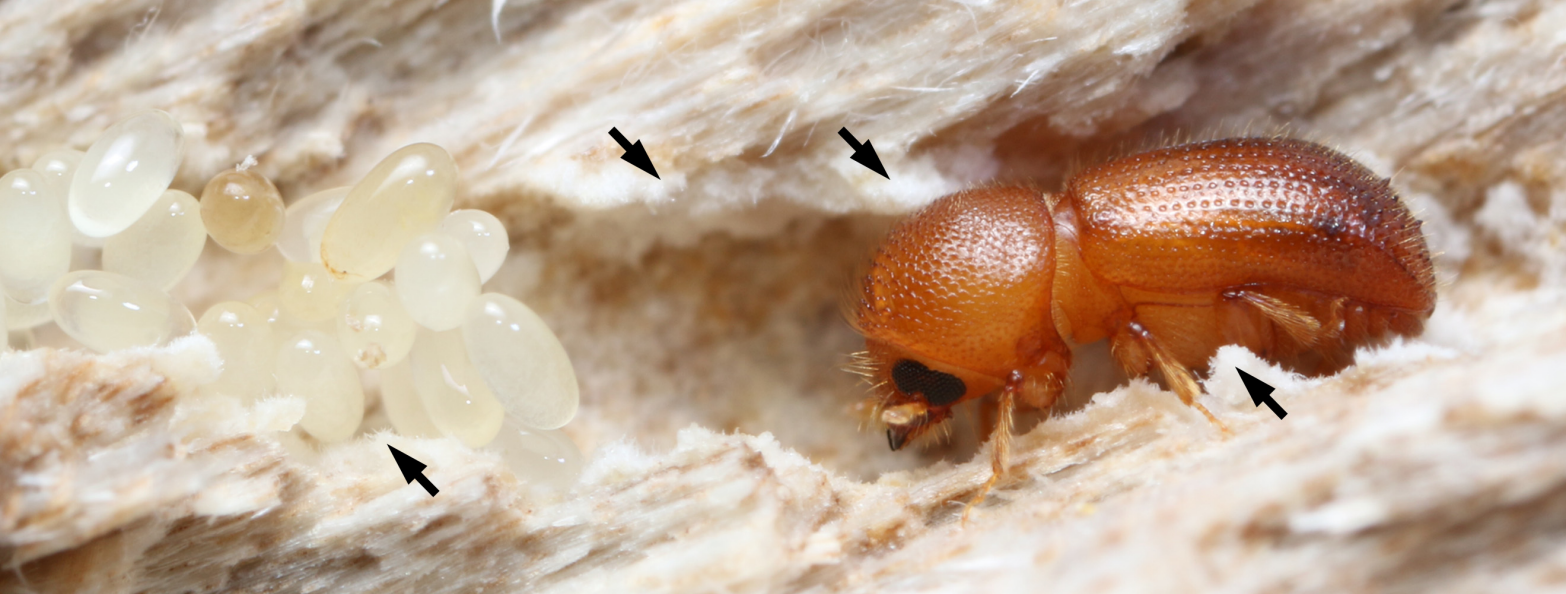
Female *Ambrosiodmus lecontei* with eggs in plant host. Note white fungal mycelial garden (arrows) in gallery.

*Ambrosiodmus* (Fig 1; [21–23]) is a genus of 80 species within the largest group of ambrosia beetles, the Xyleborini (1,200 spp.; Coleoptera: Scolytinae). The genus deserves closer scrutiny for several reasons. While its economic importance has been minimal, it is a diverse and globally distributed genus. Many species are ecologically successful and abundant throughout their range. In the twentieth century, species were increasingly established in non-native habitats via human-aided introductions. In the US alone, the originally Asiatic *A*. *rubricollis* Eichhoff [24] has been present for nearly five decades [25]. More recently, *A*. *minor* Stebbing [26], a species formerly known only from southern Asia [22], has spread across at least three southeastern states in the US [27,28].

While the majority of ambrosia fungi and ambrosia beetles depend on newly dead and relatively fresh tree tissues, *Ambrosiodmus* species appear to be able to colonize wood throughout the process of its decay, including latter stages when xylem is co-colonized by competitive wood-rot fungi. Phylogenetically, *Ambrosiodmus* is sister to *Ambrosiophilus*, an unusual genus of ambrosia beetles, many of which have lost their capacity to culture their own fungal gardens and depend on mycoclepty, or fungus theft [1]. Such unique ecological features and phylogenetic position suggest the possibility of association with fungi different than those typically associated with other ambrosia beetles, yet virtually nothing is known about the *Ambrosiodmus* symbionts. The only report on mycangia and fungal symbionts in *Ambrosiodmus* examined *A*. *rubricollis* [24] and determined the presence of preoral mycangia at the bases of the mandibles behind the labrum [29]. The mycangial content was described as globose conidia and short hyphal fragments, which yielded “white wooly mycelia only” when grown on agar.

To clarify the presence and identity of fungal symbionts of *Ambrosiodmus*, and to hypothesize upon the ecological habits and success of the genus, we characterized fungi associated with the ambrosia beetle genus and found entirely unexpected symbionts. We focused on three different *Ambrosiodmus* species: *A*. *lecontei* Hopkins [23], *A*. *minor*, and *A*. *rubricollis*, representing both the Eurasian and American fauna. We have used a comprehensive suite of complementary approaches including quantitative culturing for the main symbiont characterization, culture-independent high-throughput community sequencing for the detection of any potentially unculturable symbionts, anatomical analysis of the beetle for the characterization of the mycangia and their content, and morphological cross-validation by microscopy of the symbiont identity in the mycangia and cultured fungi.

## Materials and Methods

### Isolation and culturing of fungi from host beetles and galleries

To isolate the dominant fungal symbionts in *Ambrosiodmus*, three beetle species were sampled: *A*. *lecontei* (native to the South-Eastern US), *A*. *rubricollis* (native to Japan, but widely established in the US) and *A*. *minor* (native to SE Asia and recently established in the SE US). Female specimens of all three species were collected by light-trapping (August 2014 through April 2015) and from moribund-to-rotten wood of *Myrica cerifera* (*A*. *lecontei*), *Liquidambar styraciflua* (*A*. *rubricollis*), and *Platanus occidentalis* (*A*. *minor*), in Gainesville, Florida, USA (29° 37′ 16″ N 82° 22′ 32″ W). Collecting has been carried out on a University property and on a private property of the senior author and did not require permits. *Ambrosiodmus* are not endangered. Males have not been included in our analyses as all Xyleborini ambrosia beetle males are flightless, rare, and lack mycangia necessary for transporting symbiotic fungi [30]. Live beetles and wood sections were transported to the laboratory in clean containers with a tissue moistened with sterile water. After identification in the laboratory, whole beetles were surface-washed by vortexing for 1 min in 1 mL of sterile distilled water with one small drop (<10 μL) of Tween detergent. Beetle heads were aseptically removed, crushed, and placed in a 500 μL solution of sterile 1X phosphate buffer saline and vortexed for 30 s. Resulting solutions were diluted to 1:100 and 1:1000 concentrations, and ~100 μL of each dilutions were used to inoculate potato dextrose agar (PDA) medium (BD Difco™), which was strengthened with additional agar (5 g/L). Colony forming units (CFUs) within mycangia were estimated for each morphotype by multiplying the number of colonies on a plate by the inverse of the inoculum dilution. To determine symbionts in the ambrosial gardens, fungi were collected from *Ambrosiodmus* tunnels in *Liquidambar styraciflua* and *Platanus occidentalis* using sterile scalpels. Dominant morphotypes were quantified and purified by the methods described above.

### DNA extraction, amplification, and sequencing

Fungal DNA was isolated with Extract-N-Amp Plant PCR kits (Sigma-Aldrich Co. LLC.), with the modification of using 3% bovine serum albumin (BSA) in the place of a dilution solution. Sequences of the nuclear internal transcribed spacers ITS1-5.8S-ITS2 (ITS) and nuclear 28S ribosomal DNA (rDNA) regions were amplified with the primer combinations ITS1/ITS4 [31] and LR0R/LR5 [32], respectively, and ExTaq polymerase kits (Clontech-TaKaRa). Amplified products were visualized with SYBR® Green I Stain (Lonza Group Ltd.) on a 1% agarose gel in 1X TAE buffer. Products were purified with ExoSAP-IT (Affymetrix, Inc.) and submitted to the University of Florida Interdisciplinary Center for Biotechnology Research for Sanger sequencing. Chromatograms were assembled and inspected in Geneious (Geneious version 7.1.8).

### Phylogenetic analyses

Ribosomal DNA sequences from three representative fungal isolates were aligned to ITS and nuclear 28S rDNA sequences from additional Polyporales and Russulales taxa (S1 Table; [33,34]) in Geneious using the MAFFT alignment tool [35]. A data matrix was deposited in TreeBASE under accession number S17679. The Akaike information criterion (AIC) in jModeltest 0.1.1 [36,37] was used to select a nucleotide substitution model for the dataset. Maximum likelihood (ML) phylogenetic analyses were conducted using the suggested model parameters in GARLI 2.01 [38] and additional AIC recommended settings to determine the best tree topology. Bootstrap support values were calculated in GARLI from 100 search replicates, which were summarized with SumTrees [39]. A Bayesian phylogenetic analysis was conducted using the same AIC recommended model parameters in MrBayes 3.1.2 [40], in which two runs of four chains each were executed simultaneously from 5 000 000 generations, with sampling every 500 generations. The resulting 7501 trees retained after a burn-in of 2500 trees in SumTrees were used to compute Bayesian posterior probabilities (BPP). The new fungal species will be taxonomically described elsewhere.

### Fungal community amplicon analysis

One representative female individual of *Ambrosiodmus minor*, collected 24 November 2014 in Gainesville, FL, USA (29° 37′ 16″ N 82° 22′ 32″ W), was processed for DNA extraction and PCR amplification, as above. Amplification of nuclear 28S rDNA was targeted with primers LR0R [32] and JH-LSU-369rc (5′-CTTCCCTTTCAACAATTTCAC-3′) to yield a ~350–400 base pair (bp) product. We chose nuclear 28S rDNA rather than the more commonly used nuclear ITS rDNA “fungal barcode” because it amplifies better in many Ophiostomatales (Ascomycota: Sordariomycetes) ambrosia fungi while being sufficient for species identification in most Polyporales (previous section). ExTaq polymerase kits (Clontech-TaKaRa) were used for the amplifications. To generate an amplicon library with sequencing adaptors for Illumina MiSeq® sequencing, a double PCR protocol was used [41]. In the first PCR, the target rDNA fragments were amplified using template primers. A 1 μL aliquot of this enriched library was used as the template in the second PCR, in which fusion primers were used. The 5′ fusion primer was composed of the P5 Illumina adapter, spacer, SBS3 sequencing primer, and the template primer. The 3′ fusion primer was composed of the P7 adapter, spacer, a sample-specific 7 bp index, the SBS12 sequencing primer, and the template primer. The amplicon library was purified using the UltraClean purification kit (MoBio), visualized on an agarose gel, quantified using the QuantIT PicoGreen fluorescent dye (Life Technologies), read on an Eppendorf RealPlex qPCR thermal cycler, and diluted to a concentration comparable with other amplicons included in the sequencing run (samples not related to this project). Illumina MiSeq® sequencing was conducted at the Interdisciplinary Center for Biotechnology Research at the University of Florida. Sequence data were processed and evaluated using QIIME 1.7.0 [42]. MiSeq sequences were deposited in NCBI Sequence Read Archive under accession number SRP058537. Operational taxonomic units (OTUs) were clustered at 2% sequence similarity, and each representative sequence was identified by NCBI BLAST queries, specifically by comparison with type strain sequences, when available.

### Mycangial presence and content

Eleven females of *Ambrosiodmus lecontei* from a cryopreserved beetle depository in the Hulcr lab were examined to determine the location of mycangia. Individuals examined were collected 29 November 2010 from redbay trees (*Persea borbonia* (L.) Spreng.) near Lake Alfred, Polk County, Florida and preserved in cryotubes containing a 95% ethanol solution, which was stored at -80°C. Given the phylogenetic position of *Ambrosiodmus* within the Xyleborini with preoral mycangia [1] and a previous examination of the genus [29], the beetle heads were targeted for examination. Heads were aseptically removed from the pronotum before sectioning. Once removed, beetle heads were fixed in 10% neutral buffered formalin for 24 h and then soaked in phenol for 6 d to soften the cuticle [8]. Heads were treated with an automated tissue processor to allow desiccation of tissues and infiltration of paraffin and subsequently embedded in paraffin blocks. Heads were orientated with the most anterior end facing downward to permit transverse sectioning and simultaneous bilateral visualization of the mycangia. A Microm HM 325 rotary microtome (Walldorf, Germany) was used to cut 10-μm transverse sections. Selected slides confirmed by immediate viewing were then dried at 60°C for three days, double-stained with Harris-hematoxylin and eosin-phloxine, and examined and photographed using a Nikon Eclipse E600 compound microscope (Nikon Instruments, Melville, New York) equipped with a Nikon Digital Sight DS-Ri1 high-resolution microscope camera and Nikon NIS-Elements BR 3.2 imaging software.

### Fungal morphology on agar medium

To document the morphology of the fungal symbionts of *Ambrosiodmus*, we used six isolates that were recovered from heads of all three *Ambrosiodmus* species, as well as two isolates recovered from the galleries of *A*. *minor* and *A*. *rubricollis* (Table 1). Isolates were cultured on PDA medium and incubated at 20°C for three weeks. Hyphae were aseptically removed from the edges of the representative fungal colonies, and examined microscopically with a Nikon Eclipse 55i equipped with a ProgRes® SpeedXT core 3 camera (Jenoptik Optical Systems).

**Table 1:** Cultures of *Flavodon* cf. *flavus* and host information.

Subculture	Original culture	Host or associated beetle	Isolation source	Estimated CFU
6853	6853_white_myce	*Ambrosiodmus minor*	Head extract	1500
6855	6855_white_myce	*A*. *minor*	Head extract	1500
6860	6860_sub_white_myce	*A*. *lecontei*	Head extract	4000
7324	7324_white_myce	*A*. *lecontei*	Head extract	-
7346	7346_white_myce	*A*. *minor*	Gallery in *Platanus occidentalis*	-
7353	7326_white_myce	*A*. *rubricollis*	Head extract	30
7354	7341_white_myce	*A*. *rubricollis*	Head extract	100
7373	7325_white_myce	*A*. *rubricollis*	Gallery in *Liquidambar styraciflua*	-

## Results

### Isolation and culturing of fungi from host beetles and galleries

Individuals of each of the three *Ambrosiodmus* species contained a single fungal morphotype in at least two head extract isolation events, and estimated CFUs of the same dominant morphotype from different isolation events ranged from 10 to 4000 (Table 1). Subcultures were cryoarchived in PDA slant vials, immersed in 15% glycerol, and stored at -80°C, and deposited in the Hulcr collection at the University of Florida and at the Forestry & Agricultural Biotechnology Institute (FABI) fungal culture collection (CMW) in Pretoria, Gauteng Province, South Africa.

**Fig 2:**
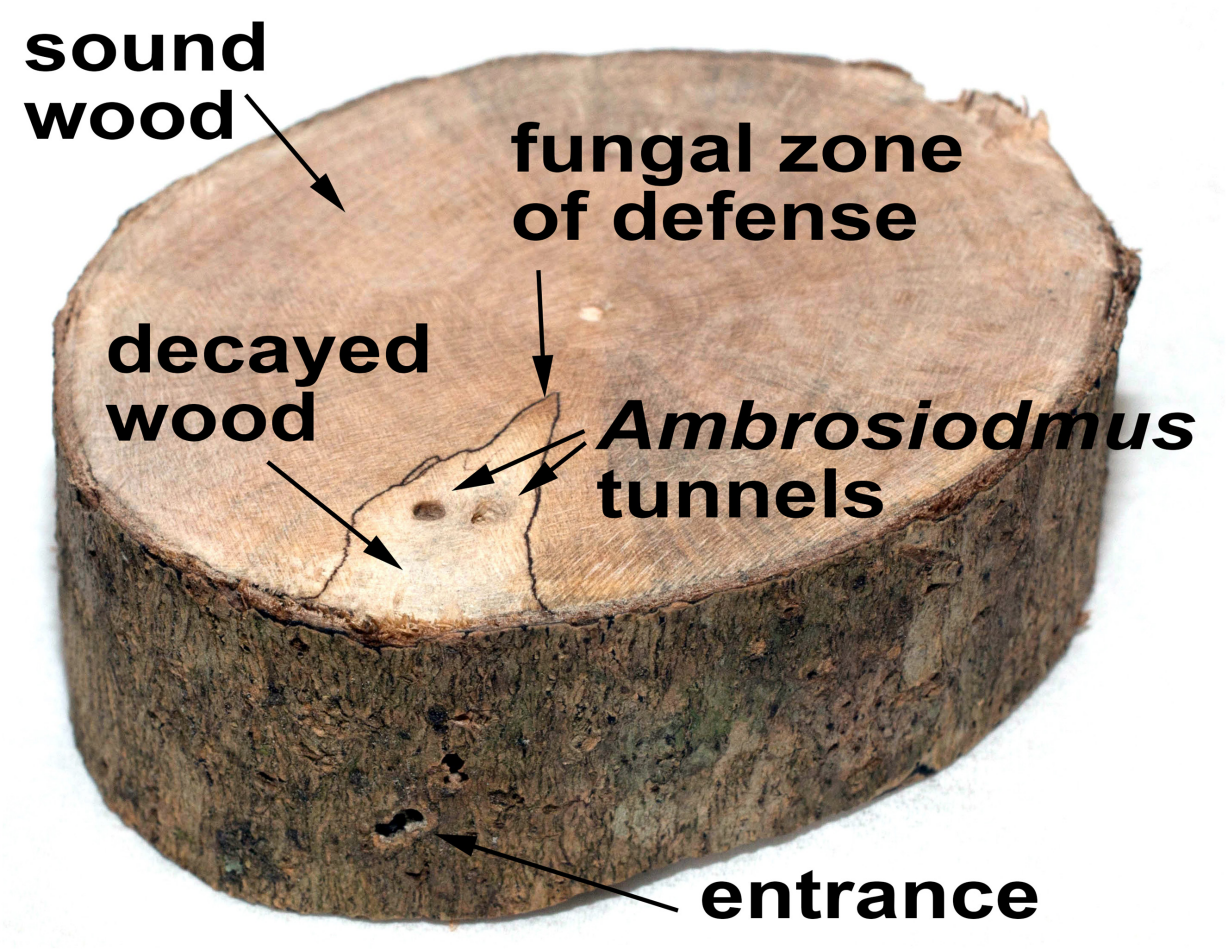
Wood decay as a result of colonization by the *Ambrosiodmus-Flavodon* symbiosis.

Active *Ambrosiodmus* galleries were routinely found in decayed wood. The wood adjacent to the *Ambrosiodmus* gallery was typically soft and spongy, and the area was surrounded by dark lines (Fig 2). These lines are a common result of antagonistic interactions among genetically distinct decay fungi in co-colonized dead wood. Other ambrosia fungi studied thus far do not form such zones of defense.

### Phylogenetic analyses

**Fig 3:**
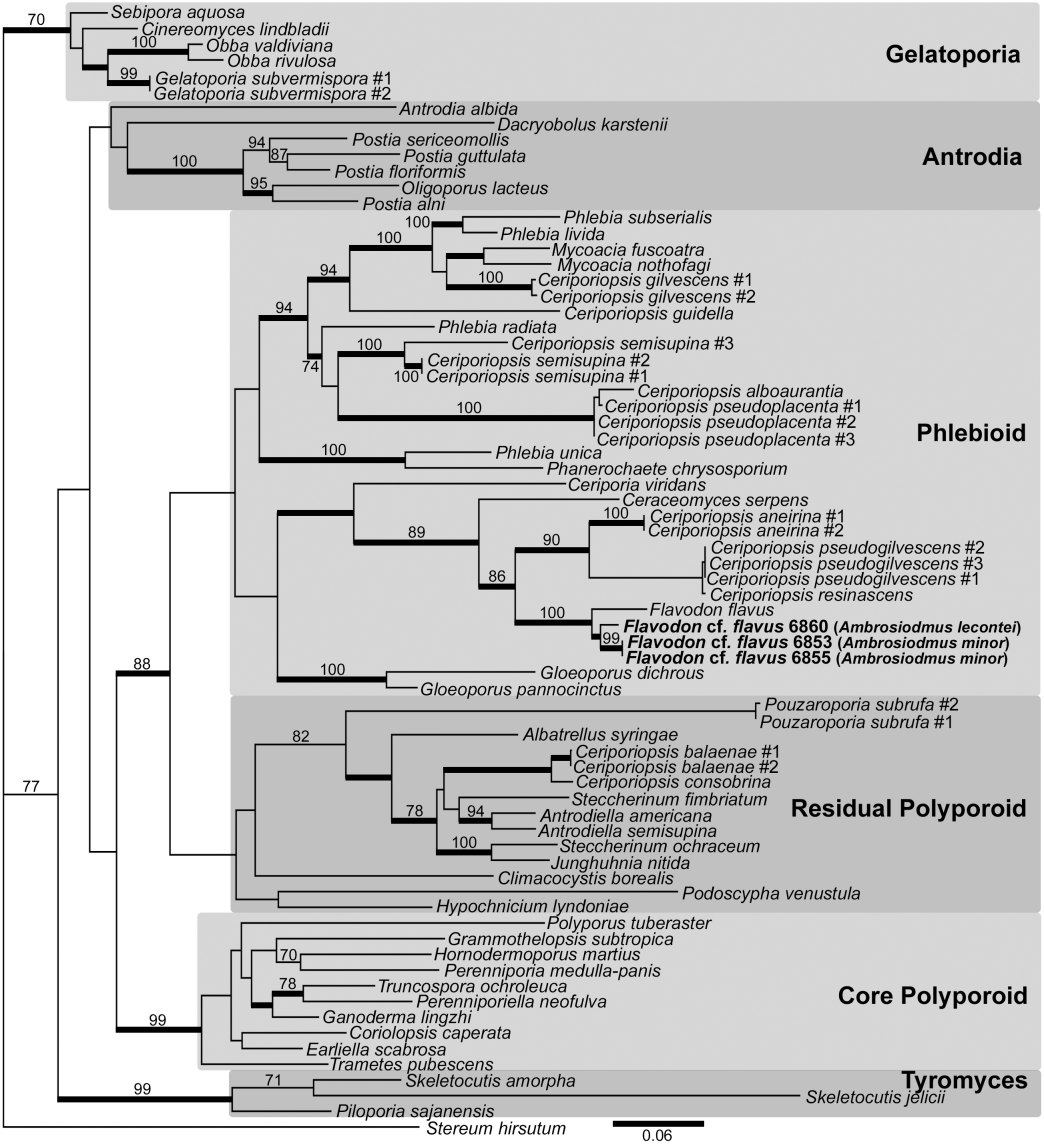
Best ML tree from GARLI analysis of combined nuclear ITS and 28S rDNA datasets. *Flavodon* cf. *flavus* isolates, with host beetle in parentheses, within phlebioid clade of Polyporales. Values at nodes indicate ≥70% ML bootstrap support, and thickened branches indicate ≥95% Bayesian posterior probability support values.

The best ML phylogeny (Fig 3) placed the representative fungal isolates from *A*. *lecontei* and *A*. *minor* heads in a monophyletic group within the phlebioid clade of the Polyporales [43] with 98% BPP but only 48% ML bootstrap support. These isolates are sister to GenBank accession JN710543 [44], identified as *Flavodon flavus* (Klotzsch) Ryvarden [45], a placement supported by 100% BPP and bootstrap value. Sequence data from all isolated cultures was deposited in GenBank accessions KR119072–KR119080 and KR871005–KR871009.

### Fungal community amplicon analysis

Sequencing of the fungal community 28S amplicon from *Ambrosiodmus minor* mycangia resulted in 355,749 sequence reads. There was a single dominant OTU, which represented 94% of the mycangial community (151345 of 160489 reads) after the removal of non-fungal sequences and OTUs corresponding to <1% of all reads. A BLAST query of the representative sequence of this OTU (5′ end of the nuclear 28S rDNA) in GenBank resulted in 26 accessions with 99% similarity, two of which were submitted as *Flavodon flavus* (accessions KP012792 and JN710543). When compared to the sequences obtained from cultured isolates, the dominant OTU was 99.48% identical (386/388 nucleotides).

### Examination of mycangia

**Fig 4:**
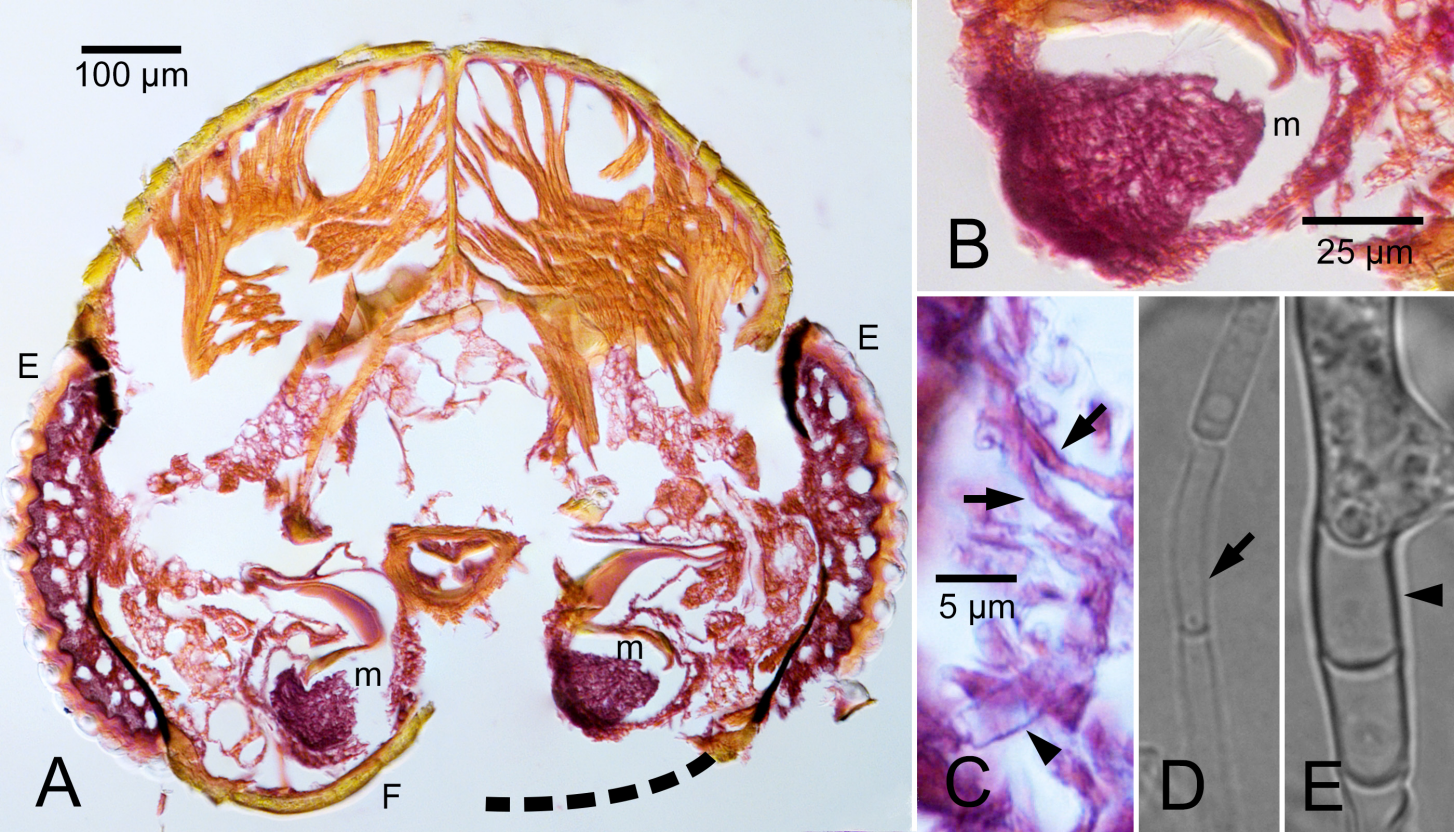
Transverse cross sections of adult female *Ambrosiodmus lecontei* heads showing position of mycangia within mouthparts. (A) Section showing entire head with paired mycangia behind the mandibles, with dashed line representing missing exoskeletal structure. (B) Compacted fungal hyphae constituting mycangial inoculum. (C) Loose hyphae from mycangial packet, with both small (arrows) and larger (arrowhead) hyphae, likely representing dimitic generative and skeletal hyphae, respectively. (D& E) *Flavodon* cf. *flavus* isolate 6860_sub_white_myce with dimitic generative (D) and skeletal (E) hyphae. Key: E, eye; F, frontal edge (accidentally damaged); m, mycangium.

Serial transverse sections from the heads of all female *A*. *lecontei* confirmed the presence of one pair of preoral mycangia at the base of the mandibles in nine of 11 beetles examined (Fig 4A and 4B). Two remaining beetle heads could not be visualized on account of sub-optimal orientation during paraffin embedding. Mycangial content was a tightly packed mass of hyphae of varying diameters (Fig 4C). No conidia or budding pseudo-mycelium were observed.

### Fungal morphology

On PDA medium, isolates 6853_white_myce, 6855_white_myce, and 6860_sub_white_myce produced dimitic hyphae, clearly of two diameters, but also with some variation in size. Smaller, generative hyphae (Fig 4D) were present at colony margins but were rapidly obscured by larger, skeletal hyphae (Fig 4E). No reproductive structures and no clamp connections were observed.

## Discussion

All analyses indicated that *Ambrosiodmus* species carry in their mycangia, and culture in their galleries, ambrosial basidiomycotan symbionts closely related to *Flavodon flavus* (Basidiomycota: Polyporales). The condition of the host trees in which the galleries of *Ambrosiodmus* were collected, and the structure of the wood surrounding the beetle gallery, indicate that the fungal symbiont is a true wood-degrading saprophyte (Fig 2). There are very few known cases in which a fungus converts degraded lignocellulose into a complete diet for its animal symbiont. Consequently, the metabolic aspects of the *Ambrosiodmus-Flavodon* symbiosis deserves further scrutiny.

The majority of the ambrosial beetle-fungus symbioses investigated thus far involve ascomycotan fungi, typically from the orders Ophiostomatales or Microascales. These relatives of plant pathogens are excellent at extracting simple nutrients from freshly dead tree tissue, but their cellulolytic capacity is limited [5]. Consequently, most ambrosia insect groups are early colonizers of dying or freshly dead trees, and are unable to utilize older wood colonized by wood-rot basidiomycotan fungi, whose enzymatic machinery for lignocellulose degradation is superior. A few basidiomycetous mutualists of wood boring insects exist. *Amylostereum* (Russulales), associated with Siricidae wood wasps, is a true saprotroph, but it does not supply the entire nutrition to its host [14]. Several phloem-feeding bark beetles and attine ants require basidiomycete mutualists for their development, but those fungi are not competitive wood-decay saprophytes [14,46]. A recent report of a basidiomycete fungus (*Antrodia*) from an unrelated ambrosia beetle *Anisandrus dispar* [13] may not refer to a nutritional symbiont, which is likely *Ambrosiella hartigii* [12,47]. The only other case in which a cellulolytic saprotroph appears to provide a complete nutrition is the *Termitomyces* mutualist of fungus-farming termites [48].

Nuclear 28S rDNA sequences of fungal isolates derived from *A*. *minor* differed from those of fungi cultured from *A*. *lecontei* and *A*. *rubricollis* by a single transitional pyrimidine mutation. Additionally, the ITS rDNA sequences from *A*. *minor* samples were identical to each other, but only 94% similar to *A*. *lecontei* samples (S1 Table). *Ambrosiodmus minor* was introduced to the US recently, compared to the long established *A*. *rubricollis* and the native *A*. *lecontei*. Taken together, these comparisons suggest the association of specific fungal strains with the established US beetle species and with the most recent immigrant beetle species.

All the isolates are approximately equally related to sequences reportedly derived from *Flavodon flavus* (Fig 3). Given the 100% supported monophyly and the matching hyphal morphology, we refer to our isolates as *Flavodon* cf. *flavus* until further analyses support their differentiation. *Flavodon flavus* is a free-living wood saprophyte described from northern Europe, a location far outside of the distribution of any *Ambrosiodmus* [44]. This may indicate that the fungal genetic markers have not yet diverged from a free-living relative, or that the association is asymmetrical, in which beetles depend on the fungus but the fungus may still be capable of an aposymbiotic lifestyle. Most symbioses between insects and basidiomycotan fungi are similarly assymetrical; this includes the Siricidae wood wasps and the bark beetles mentioned above, and also some fungus growing termites and some fungus growing attine ants [49].

We report a novel form of symbiont dispersal mode in ambrosia fungi: non-budding hyphal growth (Fig 4A and 4B). The *A*. *lecontei* mycangia appeared to be packed with hyphae of varying diameters (Fig 4C), corroborating the presence of a dimitic basidiomycotan fungus. The fungus *Flavodon flavus* was described as dimitic and lacking clamp connections [45], and this description conformed with the hyphae we observed in mycangia (Fig 4C) and from isolate 6860_sub_white_myce from *A*. *lecontei* (Fig 4D and 4E) that we molecularly characterized as *F*. cf. *flavus* (Fig 3).

The *Ambrosiodmus lecontei* mycangia are almost certainly homologous with the usual preoral mycangia in some Xyleborini ambrosia beetles; thus it appears that the unusual fungus transmission mode (hyphae) is a feature of the new fungal symbiont, not of the beetle carriers. Note that we use the term “preoral” for xyleborine mycangia that were traditionally called “mandibular”; the term “mandibular” is preoccupied by the true mandibular mycangia in *Dendroctonus* [2].

From the MiSeq analysis of fungal symbiont rDNA amplicons and sequences from cultured isolates, we posit that *Flavodon* cf. *flavus*, or a complex of cryptic species, makes up the overwhelming majority of the fungal community. It therefore seems likely that the beetle uses the fungus as its primary food source on a host plant. By an association with a saprophytic basidiomycotan fungus, it may be that the beetle genus *Ambrosiodmus* has gained access to a ubiquitous resource (dead and decaying wood) previously unavailable to any other bark or ambrosia beetle. Further study is needed to confirm this hypothesis. The approaches described above serve as a model for rapid and robust exploration and characterization of this frontier of symbiology. Given the number of ambrosia beetle and fungus species worldwide and the increasing research focus on them, we should expect more discoveries of such interactions and evolutionary innovations.

## Supporting Information

S1 Table Species of Polyporales used for phylogenetic analyses. (DOCX)
