# Correction: New Fungus-Insect Symbiosis: Culturing, Molecular, and Histological Methods Determine Saprophytic Polyporales Mutualists of *Ambrosiodmus* Ambrosia Beetles

**DOI:** 10.1371/journal.pone.0147305

**Published:** 2016-01-13

**Authors:** You Li, David Rabern Simmons, Craig C. Bateman, Dylan P. G. Short, Matthew T. Kasson, Robert J. Rabaglia, Jiri Hulcr

The first author’s name is listed incorrectly. The first author’s correct name is You Li.

The correct citation is: Li Y, Simmons DR, Bateman CC, Short DPG, Kasson MT, Rabaglia RJ, et al. (2015) New Fungus-Insect Symbiosis: Culturing, Molecular, and Histological Methods Determine Saprophytic Polyporales Mutualists of *Ambrosiodmus* Ambrosia Beetles. PLoS ONE 10(9): e0137689. doi:10.1371/journal.pone.0137689
