# Supplementary material for: New Fungus-Insect Symbiosis: Culturing, Molecular, and Histological Methods Determine Saprophytic Polyporales Mutualists of Ambrosiodmus Ambrosia Beetles
Source: PLoS One. 2015 Sep 14;10(9):e0137689. doi: 10.1371/journal.pone.0137689 (PMC4569427; doi:10.1371/journal.pone.0137689)
Supplement: S1 Table — (DOCX) [file pone.0137689.s001.docx]

Supplementary Table S1. Species of Polyporales used for phylogenetic analyses, with information on *Ambrosiodmus* symbionts in bold.

| **Species** | **Strain ID** | **Clade** | **GenBank accession no.** | |
| --- | --- | --- | --- | --- |
|  |  |  | **ITS** | **28S** |
| Ingroup: |  |  |  |  |
| *Albatrellus syringae* (Parmasto) Pouzar | Gothenburg 1488 | Residual Polyporoid | JN710607 | JN710607 |
| *Antrodia albida* (Fr.) Donk | CBS 308.82 | Antrodia | DQ491414 | AY515348 |
| *Antrodiella americana* Ryvarden & Gilb. | Gothenburg 3161 | Residual Polyporoid | JN710509 | JN710509 |
| *A. semisupina* (Berk. & M.A. Curtis) Ryvarden | FCUG 960 | Residual Polyporoid | EU232182 | EU232266 |
| *Ceraceomyces serpens* (Tode) Ginns | KHL 8478 | Phlebioid | AF090882 | AF090882 |
| *Ceriporia viridans* (Berk. & Broome) Donk | Dai 7759 | Phlebioid | KC182777 | – |
| *Ceriporiopsis alboaurantia* C.L. Zhao, B.K. Cui & Y.C. Dai | Cui 2877 | Phlebioid | KF845947 | KF845954 |
| *C. aneirina* (Sommerf.) Domański #1 | TAA 181186 | Phlebioid | FJ496683 | FJ496704 |
| *C. aneirina* #2 | Dai 12657 | Phlebioid | KF845945 | KF845952 |
| *C. balaenae* Niemelä #1 | H 7002389 | Residual Polyporoid | FJ496669 | FJ496717 |
| *C. balaenae* #2 | H 7002390 | Residual Polyporoid | FJ496668 | FJ496718 |
| *C. consobrina* (Bres.) Ryvarden | Rivoire 977 | Residual Polyporoid | FJ496667 | FJ496716 |
| *C. gilvescens* (Bres.) Domański #1 | BRNM 667882 | Phlebioid | FJ496685 | FJ496719 |
| *C. gilvescens* #2 | BRNM 710166 | Phlebioid | FJ496684 | FJ496720 |
| *C. guidella* Bernicchia & Ryvarden | HUBO 7659 | Phlebioid | FJ496687 | FJ496722 |
| *C. pseudogilvescens* (Pilát) Niemelä & Kinnunen #1 | TAA 168233 | Phlebioid | FJ496673 | FJ496702 |
| *C. pseudogilvescens* #2 | BRNM 686416 | Phlebioid | FJ496679 | FJ496703 |
| *C. pseudogilvescens* #3 | Niemelä 7447 | Phlebioid | FJ496680 | FJ496700 |
| *C. pseudoplacenta* Vlasák & Ryvarden #1 | JV050952 | Phlebioid | JN592499 | JN592506 |
| *C. pseudoplacenta* #2 | PRM 899297 | Phlebioid | JN592497 | JN592504 |
| *C. pseudoplacenta* #3 | PRM 899300 | Phlebioid | JN592498 | JN592505 |
| *C. resinascens* (Romell) Dom. | BRNM 706968 | Phlebioid | EU340896 | EU368501 |
| *C. semisupina* C.L. Zhao, B.K. Cui & Y.C. Dai #1 | Cui 10222 | Phlebioid | KF845949 | KF845956 |
| *C. semisupina* #2 | Cui 7971 | Phlebioid | KF845950 | KF845957 |
| *C. semisupina* #3 | Cui 10189 | Phlebioid | KF845951 | KF845958 |
| *Cinereomyces lindbladii* (Berk.) Jülich | KHL 12078 | Gelatoporia | FN907906 | FN907906 |
| *Climacocystis borealis* (Fr.) Kotl. & Pouzar | KH 13318 | Residual Polyporoid | JQ031126 | JQ031126 |
| *Coriolopsis caperata* (Berk.) Murrill | LE(BIN)-0677 | Core Polyporoid | AB158316 | AB158316 |
| *Dacryobolus karstenii* (Bres.) Oberw. Ex Parmasto | KHL 11162 | Antrodia | EU118624 | EU118624 |
| *Earliella scabrosa* (Pers.) Gilb. & Ryvarden | PR 1209 | Core Polyporoid | JN165009 | JN164793 |
| *Flavodon flavus* (Klotzsch) Ryvarden | FP 150441 | Phlebioid | JN710543 | JN710543 |
| ***Flavodon* cf. *flavus*** | **Hulcr 6853** | **Phlebioid** | **KR119072** | **KR119075** |
| ***Flavodon* cf. *flavus*** | **Hulcr 6855** | **Phlebioid** | **KR119073** | **KR119076** |
| ***Flavodon* cf. *flavus*** | **Hulcr 6860** | **Phlebioid** | **KR119074** | **KR119077** |
| *Ganoderma lingzhi* Sheng H. Wu, Y. Cao & Y.C. Dai | Wu 1006-38 | Core Polyporoid | JQ781858 | – |
| *Gelatoporia subvermispora* (Pilát) Niemelä #1 | BRNU 592909 | Gelatoporia | FJ496694 | FJ496706 |
| *G. subvermispora* #2 | HK 20823 | Gelatoporia | FN907911 | FN907911 |
| *Gloeoporus pannocinctus* (Romell) J. Erikss. | BRNM 709972 | Phlebioid | EU546099 | FJ496708 |
| *G. dichrous* (Fr.) Bres. | KHL 11173 | Phlebioid | EU118627 | EU118627 |
| *Grammothelopsis subtropica* B.K. Cui & C.L. Zhao | Cui 9041 | Core Polyporoid | JQ845096 | JQ845099 |
| *Hornodermoporus martius* (Berk.) Teixeira | MUCL 41677 | Core Polyporoid | FJ411092 | FJ393859 |
| *Hypochnicium lyndoniae* (D.A. Reid) Hjortstam | NL 041031 | Residual Polyporoid | JX124704 | JX124704 |
| *Junghuhnia nitida* (Pers.) Ryvarden | KHL 11903 | Phlebioid | EU118638 | EU118638 |
| *Mycoacia fuscoatra* (Fr.) Donk | KHL 13275 | Phlebioid | JN649352 | JN649352 |
| *M. nothofagi* (G. Cunn.) Ryvarden | KHL 13750 | Phlebioid | GU480000 | GU480000 |
| *Obba rivulosa* (Berk. & M.A. Curtis) Miettinen & Rajchenb. | KCTC 6892 | Gelatoporia | FJ496693 | FJ496710 |
| *O. valdiviana* (Rajchenb.) Miettinen & Rajchenb. | FF 503 | Gelatoporia | HQ659235 | HQ659235 |
| *Oligoporus lacteus* (Fr.) Gilb. & Ryvarden | X 1391 | Antrodia | KC595939 | KC595939 |
| *Perenniporia medulla-panis* (Jacq.) Donk | MUCL 49581 | Core Polyporoid | FJ411088 | FJ393876 |
| *Perenniporiella neofulva* (Lloyd) Decock & Ryvarden | MUCL 45091 | Core Polyporoid | FJ411080 | FJ393852 |
| *Phanerochaete chrysosporium* Burds. | BKM-F-1767 | Phlebioid | HQ188436 | GQ470643 |
| *Phlebia livida* (Pers.) Bres. | FCUG 2189 | Phlebioid | AF141624 | AF141624 |
| *P. radiata* Fr. | UBCF 19726 | Phlebioid | HQ604797 | HQ604797 |
| *P. subserialis* (Bourdot & Galzin) Donk | FCUG 1434 | Phlebioid | AF141631 | AF141631 |
| *P. unica* (H.S. Jacks. & Dearden) Ginns | KHL 11786 | Phlebioid | EU118657 | EU118657 |
| *Piloporia sajanensis* (Parmasto) Niemelä | Mannine 2733a | Tyromyces | HQ659239 | HQ659239 |
| *Podoscypha venustula* (Speg.) D.A. Reid | CBS 65684 | Residual Polyporoid | JN649367 | JN649367 |
| *Polyporus tuberaster* (Jacq. ex Pers.) Fr. | CulTENN 8976 | Core Polyporoid | AF516598 | AJ488116 |
| *Postia alni* Niemelä & Vampola | X 1400 | Antrodia | KC595932 | KC595932 |
| *P. floriformis* (Quél.) Jülich | Gothenburg 4120 | Antrodia | KC595937 | KC595937 |
| *P. guttulata* (Peck ex. Sacc.) Jülich | KHL 11739 | Antrodia | EU118650 | EU118650 |
| *P. sericeomollis* (Romell) Jülich | X 1332 | Antrodia | KF112878 | KF112878 |
| *Pouzaroporia subrufa* (Ellis & Dearn.) Vampola #1 | BRNM 710164 | Residual Polyporoid | FJ496661 | FJ496723 |
| *P. subrufa* #2 | BRNM 710172 | Residual Polyporoid | FJ496662 | FJ496724 |
| *Sebipora aquosa* Miett. | Miettinen 8680 | Gelatoporia | HQ659240 | HQ659240 |
| *Skeletocutis amorpha* (Fr.) Kotl. & Pouzar | Miettinen 11038 | Tyromyces | FN907913 | FN907913 |
| *S. jelicii* Tortič & A. David | H 6002113 | Tyromyces | FJ496690 | FJ496727 |
| *Steccherinum fimbriatum* (Pers.) J. Erikss. | KHL 11905 | Residual Polyporoid | EU118668 | EU118668 |
| *S. ochraceum* (Pers.) Gray | KHL 11902 | Residual Polyporoid | JQ31130 | JQ31130 |
| *Trametes pubescens* (Schumach.) Pilát | PRM 900586 | Core Polyporoid | AY684173 | AY855906 |
| *Truncospora ochroleuca* (Berk.) Pilát | MUCL 39726 | Core Polyporoid | FJ411098 | FJ393865 |
|  |  |  |  |  |
| Outgroup: |  |  |  |  |
| *Stereum hirsutum* (Willd.) Pers. | NBRC 6520 |  | AB733150 | AB733325 |
